# Japanese Health Information Technology Usability Evaluation Scale for Sexually Transmitted Infection–Related Chatbots: Development and Psychometric Validation Study

**DOI:** 10.2196/90483

**Published:** 2026-07-23

**Authors:** Tomoko Hato, Hirono Ishikawa, Kense Todo, Keisuke Harada, Atsushi Yoshikawa, Yoshiharu Fukuda, Rebecca Schnall

**Affiliations:** 1 Graduate School of Public Health, Teikyo University, 2-11-1 Kaga, Itabashi-ku, Tokyo, 173-8605, Japan, 81 3-3964-1211; 2 College of Informatics, Kanto Gakuin University, Kanagawa, Japan; 3 School of Computing, Institute of Science Tokyo, Tokyo, Japan; 4 School of Nursing, Columbia University, New York, NY, United States

**Keywords:** mobile health, mHealth, chatbot, usability, translation, validation, sexually transmitted infection, STI, artificial intelligence, conversational AI

## Abstract

**Background:**

The rapid expansion of mobile technology has accelerated the integration of health applications and conversational AI into clinical and public health practices. To ensure these tools are effective and sustainable, usability evaluations and early user engagement during development are essential. The Health Information Technology Usability Evaluation Scale (Health-ITUES) is a validated and flexible usability assessment instrument that is available in multiple languages and applicable across diverse contexts. However, a Japanese version of this scale has not yet been developed.

**Objective:**

This study aimed to translate and validate a Japanese version of the Health-ITUES, customized for a sexually transmitted infection (STI)–related chatbot, and to support the usability assessment of emerging mobile health tools in Japan.

**Methods:**

We developed a Japanese version of the Health-ITUES using a chatbot under development as a consultation tool for young women regarding STIs. First, the original scale was customized to reflect the chatbot’s specific purpose and intended usage context. Following established translation guidelines, we conducted forward translation from English to Japanese, back translation, expert review, and reconciliation. We then evaluated the reliability and validity of the Japanese version in a sample of 301 young women.

**Results:**

The Japanese version of the Health-ITUES demonstrated high internal consistency (Cronbach α=0.85‐0.98). Confirmatory factor analysis supported acceptable construct validity (root mean square error of approximation is 0.10, comparative fit index>0.90). Additionally, the Health-ITUES scores showed strong correlations with satisfaction and usage intention for the tool (*r*=0.779 and 0.797, respectively).

**Conclusions:**

The Japanese version of the Health-ITUES provides initial evidence of reliability and validity in an STI-related scenario among young women and may facilitate more rigorous usability evaluations of mHealth and conversational AI tools in Japan.

## Introduction

In recent years, the widespread adoption of smartphones and tablets has led to a significant increase in their daily usage. According to the Ministry of Internal Affairs and Communications’ 2024 survey on information and communication usage trends, the individual smartphone ownership rate in Japan has reached 80.5%, with over 90% penetration among individuals aged 13 to 69 years [1]. As information and communication technologies continue to evolve, mobile health (mHealth), which uses smartphones and portable devices for health care, has been rapidly adopted worldwide. mHealth is already applied across various domains, such as health management, patient education, and disease prevention, and is expected to improve health care accessibility and support self-management practices [2-4].

However, several mHealth technologies have become publicly available without sufficient validation of their design, development, or evaluation processes [5]. Although acknowledging the potential of mobile-based interventions in strengthening health systems, the World Health Organization (WHO) emphasizes the need for careful assessment of their acceptability, effectiveness, feasibility, and equity in its guidelines on digital health interventions [6]. The effectiveness and sustainability of mHealth interventions depend not only on technical performance but also on user-centered evaluations of usability and acceptability [7]. Accordingly, evaluating mHealth technologies requires a more comprehensive approach beyond measuring clinical outcomes alone. It is essential to assess their usability and acceptability from the user’s perspective and incorporate user feedback from the early stages of development. In recent years, the emergence of conversational AI systems, such as ChatGPT, has further expanded the scope of mHealth. Although these systems still face challenges related to information reliability and data security, they offer the potential to facilitate interactive bidirectional communication with users, thereby contributing to behavioral change, health education, and self-management support [8,9]. Previous studies have shown that, by demonstrating empathy and a sense of familiarity, chatbots can improve health outcomes in stigmatized areas, such as sexually transmitted infections (STIs) and mental health [10-13]. However, their effectiveness may be limited if they fail to recognize user characteristics, expectations, or levels of understanding [14,15]. Even when large language model–based conversational AIs, such as ChatGPT, provide highly accurate responses, the quality of user interaction can significantly influence the ability to extract relevant information. In some cases, these interactions may even lead to misinterpretations or hinder users’ health-related decision-making [16]. Therefore, usability evaluation has become a critical component in the design and assessment of such systems.

Usability is defined as the extent to which users can effectively and efficiently achieve specific goals with satisfaction. Several usability assessment tools have been developed to evaluate these aspects [17]. Early examples include the System Usability Scale (SUS) [18] and the Post-Study System Usabiity Questionnaire (PSSUQ) [19], which were originally designed for general IT systems. However, prior research has indicated that general-purpose usability measures may not adequately capture the specific requirements of health care contexts [20]. Tools such as the Mobile App Rating Scale (MARS/uMARS) [21,22], the mHealth App Usability Questionnaire (MAUQ), and the Health Information Technology Usability Evaluation Scale (Health-ITUES) [23] have been developed for mHealth-specific applications.

Health-ITUES was originally developed using a web-based communication system designed for managing nursing staff schedules [23]. Since then, it has been applied to evaluate the usability of various health technologies, including mobile applications for individuals living with HIV, apps for managing dysmenorrhea, and integrated care systems for older adults [24-27]. The Health-ITUES is one of the few instruments whose psychometric properties have been validated in accordance with the COSMIN (Consensus-Based Standards for the Selection of Health Measurement Instruments) guidelines [17]. Unlike general usability scales (eg, SUS), the Health-ITUES is designed specifically for health information technologies and is grounded in the Health IT Usability Evaluation Model (Health-ITUEM) framework, enabling the evaluation of clinically meaningful usability constructs [23,27]. It maintains conceptual comparability across different contexts while allowing item-level customization to suit specific tasks and objectives within health care systems [28,29].

To date, the Health-ITUES has been translated and validated in several languages, such as Korean [25] and Chinese [26], demonstrating its cross-cultural applicability. However, no validated Japanese version is currently available. We selected Health-ITUES for this study because its health-IT–oriented design, validated psychometric robustness, and flexibility for context-specific item adaptation make it particularly well suited for evaluating an STI-related chatbot scenario targeting young women [25,27]. By developing and validating a Japanese version tailored to STI-related consultations, this study aimed to provide a rigorous and contextually relevant tool for evaluating the usability of emerging mHealth and conversational AI tools in Japan.

## Methods

### Modification of the Health-ITUES

The Health-ITUES consists of 20 items and can be customized according to the characteristics of the device and the intended use. Originally, it was developed based on the Technology Acceptance Model (TAM) to evaluate the usability of a web-based scheduling system for nursing staff [23]. Subsequently, Schnall et al [27] further validated the scale using a self-management application for individuals living with HIV, focusing on the evaluation of self-management mHealth applications.

In this study, we customized the Health-ITUES for young women experiencing anxiety related to STIs, based on the validated versions used among HIV-positive individuals in the United States and women with menstrual discomfort in South Korea. Prior to initiating the translation and adaptation process, we obtained permission from the original author to develop a Japanese version of the Health-ITUES.

### Japanese Version of the Health-ITUES

The translation process adhered to established guidelines for scale translation [30] and followed the sequence as described in the following sections.

#### Forward Translation and Reconciliation

Two researchers (a physician and a health communication specialist) independently translated the original English version into Japanese. Subsequently, they discussed and reconciled their translations. A health communication research group, comprising physicians and health psychology experts, reviewed the preliminary Japanese version to ensure clarity, cultural appropriateness, and linguistic accuracy.

#### Back Translation

Two professional medical translators independently translated the Japanese version of the questionnaire back into English. The translations were then compared to confirm consistency and accuracy.

#### Back-Translation Review

The authors reviewed the back-translated version by retranslating it into Japanese and comparing it with the initial Japanese draft to identify discrepancies.

#### Original Author Review

After completing both the forward and backward translations, the original author reviewed the English version of the Japanese Health-ITUES to confirm its content validity.

#### Cognitive Debriefing

We conducted cognitive debriefing interviews to assess the clarity and contextual appropriateness of the Japanese version. Linguistic validation guidelines recommend a small sample from the target population, with a Japanese guideline suggesting approximately 5 to 8 participants [30-32]. In this study, 6 female students studying at a university without a medical background were interviewed using the Japanese Health-ITUES. They were asked whether any items were difficult to understand or caused hesitation when responding.

### Reliability and Validity

We conducted an online survey using a chatbot under development by the authors to evaluate the reliability and validity of the Japanese version of the Health-ITUES. The chatbot was designed to alleviate anxiety related to STIs among young women and to guide them toward appropriate consultation services and testing resources. Internal consistency reliability was assessed using the Cronbach α coefficient. Construct validity was examined through confirmatory factor analysis (CFA), and criterion-related validity was evaluated by examining correlations with user satisfaction and intention to use.

### Participants

Participants were women aged 18 to 29 years registered with a Japanese research agency (Asmarq Inc). According to national infectious disease surveillance data [33], reported syphilis cases—one of the major STIs—among women in their 20s have increased substantially since 2020, making this demographic particularly relevant for the chatbot’s intended use. Eligibility criteria included the ability to communicate in Japanese, access the survey URL using personal computers or smartphones, and have had at least one prior sexual experience. Individuals who were health care professionals, medical students, or already engaged with STI-related medical services or consultation centers were excluded from the study.

### Sample Size

To ensure adequate statistical power in scale validation studies, a commonly used rule of thumb is to recruit approximately 10 participants per item [32,34]. Given that the Health-ITUES consists of 20 items, we estimated the minimum required sample size to be 200 participants. Assuming a 20% dropout rate, the target sample size was set at 250 participants.

### Procedure

Participants were recruited through a research agency. The agency randomly selected 57,185 women within the target age range from its registered panel and sent email invitations to participate in the study. Only those who provided informed consent via an embedded survey link were directed to the main questionnaire. The survey was terminated once the number of responses exceeded the target sample size. Data were collected between June 18, 2025, and June 20, 2025. A total of 301 valid responses were obtained.

Participants were informed via a web-based survey interface that the study involved interacting with a chatbot based on a hypothetical scenario (ie, their partner suddenly informed them of symptoms suggestive of an STI). Instructions on how to use the chatbot were also provided. Initially, participants completed prechatbot questions on age, region of residence, occupation, educational background, relationship status, frequency of conversational AI use, and digital health literacy [35]. They then accessed the chatbot interface through a URL embedded in the survey and interacted with it according to the scenario. After the interaction, participants returned to the survey and completed postchatbot questions assessing satisfaction, intention to use, and the Japanese version of the Health-ITUES.

### Survey Items

The survey included the following items:

- Participant demographics: the demographics consisted of age, region of residence, educational background, occupation, and relationship status.
- Frequency of conversational AI use: items were rated on a 4-point scale ranging from “never” to “almost daily.”
- Digital health literacy: this was measured using 15 items from the Japanese version of the Digital Health Literacy Instrument [35], excluding the subscales for operational and navigational skills.
- Postchatbot satisfaction: items were rated on a 5-point scale ranging from “very satisfied” to “not at all satisfied.”
- Intention to use: this item was assessed with the question, “Would you consider using this chatbot if you were experiencing anxiety or concerns about STIs?” and rated on a 5-point scale ranging from “strongly agree” to “strongly disagree.”
- Usability: usability was evaluated using the 20-item Japanese version of the Health-ITUES (Table 1).

**Table 1.** Health-ITUES^a^ [27] and Japanese Health-ITUES.

Items in the Health-ITUES [27]	Items in the Japanese version of Health-ITUES
Impact	
1. I think mVIP^b^ would be a positive addition for persons living with HIV.	1. I think this chatbot is helpful for young women who have concerns or symptoms related to sexually transmitted infections.
2. I think mVIP would improve the quality of life of persons living with HIV.	2. I think this chatbot will improve the quality of life of young women who have concerns or symptoms related to sexually transmitted infections.
3. mVIP is an important part of meeting my information needs related to symptom self-management.	3. This chatbot plays an important role in helping me get the information I need about concerns and symptoms related to sexually transmitted infections.
Perceived usefulness	
4. Using mVIP makes it easier to self-manage my HIV-related symptoms.	4. Using this chatbot makes it easier for me to get information about sexually transmitted infections and ease my concerns about them.
5. Using mVIP enables me to self-manage my HIV-related symptoms more quickly.	5. Using this chatbot allows me to get information about sexually transmitted infections faster and ease my concerns about them.
6. Using mVIP makes it more likely that I can self-manage my HIV-related symptoms.	6. Using this chatbot increases the likelihood of me getting information about sexually transmitted infections and easing my concerns about them.
7. Using mVIP is useful for self-management of HIV-related symptoms.	7. Using this chatbot is helpful in getting information about sexually transmitted infections and easing my concerns about them.
8. I think mVIP presents a more equitable process for self-management of HIV-related symptoms.	8. I think this chatbot equally helps everyone get information about sexually transmitted infections and ease their concerns about them.
9. I am satisfied with mVIP for self-management of HIV-related symptoms.	9. I am happy to use this chatbot to get information about sexually transmitted infections and ease my concerns about them.
10. I self-manage my HIV-related symptoms in a timely manner because of mVIP.	10. Thanks to this chatbot, I will be able to get information about sexually transmitted infections and ease my concerns about them at the right time.
11. Using mVIP increases my ability to self-manage my HIV-related symptoms.	11. Using this chatbot improves my ability to get information about sexually transmitted infections and ease my concerns about them.
12. I am able to self-manage my HIV-related symptoms whenever I use mVIP.	12. Using this chatbot allows me to get information about sexually transmitted infections and ease my concerns about them at any time.
Perceived ease of use	
13. I am comfortable with my ability to use mVIP.	13. I can feel comfortable using this chatbot.
14. Learning to operate mVIP is easy for me.	14. I can easily learn how to use this chatbot.
15. It is easy for me to become skillful at using mVIP.	15. It is easy for me to master using this chatbot.
16. I find mVIP easy to use.	16. I think this chatbot is easy to use.
17. I can always remember how to log on to and use mVIP.	17. I remember how to access this chatbot and how to use it.
User control	
18. mVIP gives error messages that clearly tell me how to fix problems.	18. This chatbot displays error messages that clearly indicate how to resolve any problems with it.
19. Whenever I make a mistake using mVIP, I can recover easily and quickly.	19. If I make a mistake on how to use this chatbot while using it, I can easily undo it.
20. The information (such as online help, on-screen messages, and other documentation) provided with mVIP is clear.	20. The information provided by this chatbot (online help, messages, etc) is easy to understand.

a Health-ITUES: Health Information Technology Usability Evaluation Scale.

b mVIP: HIV self-management app.

### Statistical Analysis

We calculated the mean scores and SDs for the overall Japanese version of the Health-ITUES and its subscales (impact, perceived usefulness, ease of use, and user control). Ceiling and floor effects (acceptable if <15% at maximum and minimum scores) were examined. We assessed reliability through internal consistency, using the Cronbach α coefficient and composite reliability (CR). Convergent validity was evaluated based on the average variance extracted (AVE). To assess construct validity, a CFA was conducted according to the original 4-factor model. Model fit was evaluated using fit indices, including the Goodness-of-Fit Index (GFI), Comparative Fit Index (CFI), Tucker-Lewis Index (TLI), root mean square error of approximation (RMSEA), and standardized root mean square residual (SRMR). Criterion-related validity was assessed by examining the correlation coefficients between Health-ITUES scores and conceptually related variables, namely user satisfaction and intention to use the chatbot. All statistical tests were conducted at a 5% significance level using IBM SPSS Statistics version 29.0 and AMOS version 31.0.

### Ethical Considerations

This study was approved by the Research Ethics Committee for human participants at Teikyo University (approval numbers: Tei-Rin 24‐114, dated January 30, 2024, and Tei-Rin 25‐006, dated May 8, 2025). Participants provided informed consent via an embedded survey link. Participants received a token of appreciation, equivalent to 200 Japanese yen (1 Japanese yen=US $0.006 as of May 8, 2025) in points, for their participation in the study.

## Results

### Pilot Test

We conducted a pilot test with 6 female university students aged 18 to 22 years, recruited through convenience sampling. All participants were native Japanese speakers and nonmedical majors. They completed the Japanese version of the Health-ITUES and were subsequently interviewed regarding the clarity of language, ease of response, and confidence in their responses. One participant commented, “There were no particular difficulties in responding, but because the chatbot was accessed via a URL rather than through a login, it might be better to revise that wording.” Based on this feedback, we revised item 17 to clarify that the chatbot was accessed via a URL in this study.

### Demographic Characteristics

Table 2 presents the demographic characteristics of the 301 participants. Their mean age was 26.3 (SD 2.1) years. Although more than half of the participants were company employees, the sample included a diverse range of occupations, such as students, homemakers, and part-time workers. The most common frequency of conversational AI use was “occasionally” (n=109, 36.2%), followed by “never,” “rarely,” and “almost daily,” each accounting for approximately 20% of responses. When “occasionally” and “almost daily” users are combined, approximately 60% (n=177) of participants reported using conversational AI to some extent, which is consistent with national data indicating that around 50% of young people in Japan use such technologies [36].

**Table 2.** Participant characteristics (N=301).

Characteristics	Values
Age (y), mean (SD)	26.36 (2.12)
Digital Health Literacy Instrument, mean (SD)	2.83 (0.44)
Region of residence, n (%)	
Hokkaido	9 (3.0)
Tohoku	14 (4.7)
Kanto	129 (42.9)
Chuubu	39 (13.0)
Kinki	62 (20.6)
Chuugoku/Shikoku	25 (8.3)
Kyushuu/Okinawa	23 (7.6)
Occupation, n (%)	
Company employee or government worker	181 (60.1)
Self-employed	4 (1.3)
Homemaker	33 (11.0)
Part-time worker or temporary worker	47 (15.6)
Student	9 (3.0)
Other	27 (9.0)
Educational background, n (%)	
Junior high school	7 (2.3)
High school	79 (26.2)
Vocational school	36 (12.0)
Junior college	14 (4.7)
College or university	153 (50.8)
Graduate school or higher	9 (3.0)
Other	3 (1.0)
Frequency of conversational AI use, n (%)	
Never	62 (20.6)
Rarely	62 (20.6)
Occasionally	109 (36.2)
Almost daily	68 (22.6)
Relationship status, n (%)	
Has a specific partner	241 (80.1)
Has a nonspecific partner	15 (5.0)
No partner	43 (14.3)
Other	2 (0.7)

The average score on the 15-item Digital Health Literacy Scale (DHLS) was 2.8 (SD 0.4). This result aligns closely with the findings from a study of Japanese university students, which reported an average score of 2.9 (SD 0.4) on the 21-item DHLS [37]. These findings indicate that the study sample is broadly representative of young Japanese individuals in terms of digital health literacy.

### Overall Scores

The Health-ITUES consists of 20 items across 4 subscales (impact: 3 items, perceived usefulness: 9 items, ease of use: 5 items, and user control: 3 items), each rated on a 5-point Likert scale (1=“strongly disagree” to 5=“strongly agree”). Satisfaction was assessed on a 5-point scale (1=“not satisfied at all” to 5=“very satisfied”), along with intention to use (1=“not at all willing to use” to 5=“very willing to use”). The overall mean score for the Japanese version of Health-ITUES was 3.4 (SD 0.9), whereas user satisfaction was 3.1 (SD 1.2) and intention to use was 3.2 (SD 1.2; Table 3). Among the subscales, user control showed slightly lower mean scores, although no substantial differences were observed. No ceiling or floor effects were detected.

**Table 3.** Mean scale and internal scale consistency scores (N=301).

Score	Mean (SD)	Cronbach α	CR^a^	AVE^b^
Health-ITUES^c^ (overall)	3.427 (0.890)	0.976	—^d^	—
Health-ITUES impact	3.517 (1.012)	0.953	0.948	0.860
Health-ITUES usefulness	3.450 (0.959)	0.976	0.974	0.806
Health-ITUES ease of use	3.443 (0.980)	0.922	0.908	0.667
Health-ITUES control	3.241 (0.960)	0.849	0.830	0.624
User satisfaction	3.080 (1.169)	—	—	—
Intention to use	3.159 (1.209)	—	—	—

a CR: composite reliability.

b AVE: average variance extracted.

c Health-ITUES: Health Information Technology Usability Evaluation Scale.

d Not applicable.

### Internal Consistency Reliability

The internal consistency and convergent validity were evaluated, with Cronbach α of more than 0.8, CR exceeding 0.70, and AVE exceeding 0.50. As shown in Table 3, Cronbach α coefficients for the subscales ranged from 0.85 to 0.98, indicating high reliability beyond the commonly accepted cutoff of 0.80. The impact and perceived usefulness subscales had particularly high α values (>0.95). The overall Cronbach α for all 20 items was 0.976, which may be attributed to the large number of items in the usefulness subscale and high intersubscale correlations. Descriptive statistics of the 20 items (mean, SD, skewness, kurtosis, and item-total correlations) are provided in Multimedia Appendix 1.

### Construct Validity

As the conceptual validity of the original Health-ITUES had already been established, we performed a CFA using maximum likelihood estimation based on the original 4-factor structure. The analysis provided standardized factor loadings to assess the strength of the relationship between items and factors, along with model fit indices.

**Figure 1.**
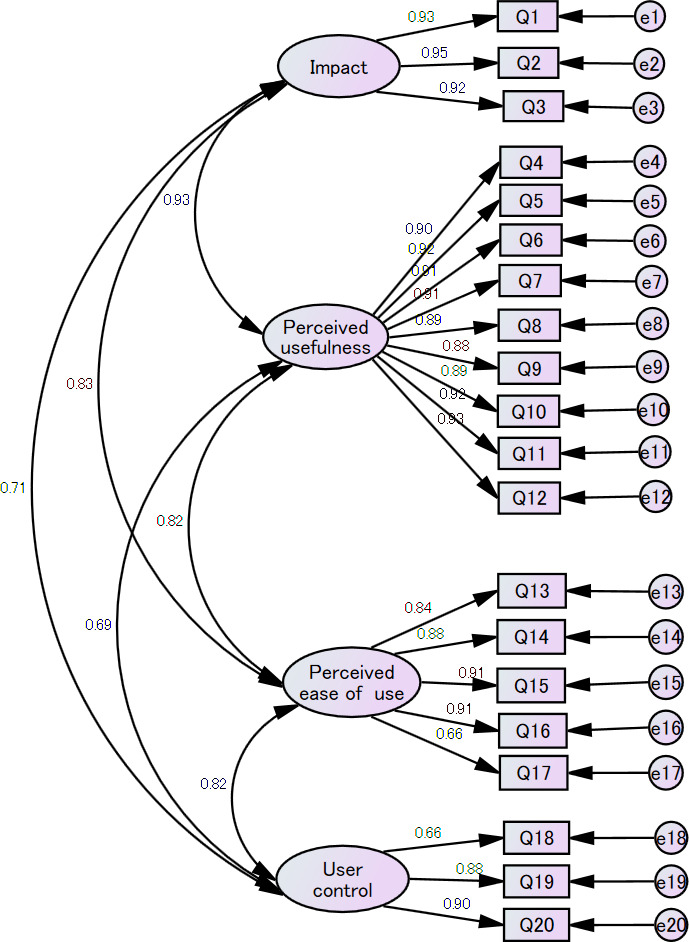
Confirmatory factor analysis path diagram of the Japanese Health-ITUES: Health Information Technology Usability Evaluation Scale.

The path diagram is shown in Figure 1, and Table 4 presents the model fit indices. The CFA results indicated an adequate model fit (CFI=0.94, TLI=0.93, and SRMR=0.032). However, the GFI was relatively low (0.8), and the RMSEA was 0.100, representing the upper limit of the acceptable range [26].

**Table 4.** Confirmatory factor analysis model fit indices for the Health-ITUES^a^.

Fit index	CFI^b^	TLI^c^	RMSEA^d^	SRMR^e^	GFI^f^	Chi-square/*df*
Value	0.935	0.925	0.100	0.0319	0.799	660.92/164
Recommended threshold (desirable)	≥0.90 (≥0.95)	≥0.90 (≥0.95)	≤0.08 (≤0.05)	≤0.08 (≤0.05)	≥0.90	<3.00

a Health-ITUES: Health Information Technology Usability Evaluation Scale.

b CFI: Comparative Fit Index.

c TLI: Tucker-Lewis Index.

d RMSEA: root mean square error of approximation.

e SRMR: standardized root mean square residual.

f GFI: Goodness-of-Fit Index.

Intersubscale correlations ranged from 0.69 to 0.93, indicating strong relationships. In particular, perceived usefulness showed a higher correlation with impact, which is consistent with the findings of previous research [25,26,29].

We examined criterion-related validity by calculating the correlation coefficients between Health-ITUES scores and conceptually related variables, namely user satisfaction and intention to use. The correlations were 0.779 and 0.797, respectively, indicating a strong positive association (*P*<.001).

## Discussion

### Principal Findings

The emergence of generative AI has expanded the potential of mHealth. Chatbots are increasingly recognized as promising tools for supporting STI prevention and sexual health education among young people, and their role is expected to grow [11,38-41]. At the same time, usability—including user satisfaction and ease of use—remains critical for promoting continued engagement and facilitating behavior change [39,40].

The instrument selected for this study—the Health-ITUES—was developed as a user-centered evaluation scale based on the Health-ITUEM, which itself was derived from the widely used TAM and specifically adapted for health care technologies [23,28].

This study developed a Japanese version of the Health-ITUES and examined its reliability and validity using a chatbot currently under development as a consultation tool for young women with concerns about STIs. Because the original scale was designed to evaluate an app for adults living with HIV, we adapted the item wording to align with the chatbot’s purpose and functions rather than conducting a direct translation.

In this study, we confirmed that the 4-factor structure of the Health-ITUES—impact, usefulness, ease of use, and user control—was largely retained in the context of a Japanese STI-related chatbot.

The translation procedures followed established guidelines, including forward translation by 2 experts in medicine and health communication, review by a multidisciplinary team of physicians and health communication experts, and a back-translation performed by a medical translation specialist. Cognitive debriefing and a final review by one of the authors, who validated the original Health-ITUES, ensured the cultural appropriateness and conceptual consistency of the translated version.

The customized Health-ITUES demonstrated adequate reliability and convergent validity for evaluating the usability of the STI-related scenario chatbot, and the main model-fit indices were satisfactory. In the CFA, although the RMSEA was slightly higher, the overall model fit was still considered acceptable because the other indices indicated a generally adequate level of fit [26]. Strong positive correlations between Health-ITUES scores and both user satisfaction and intention to use further supported criterion validity. These findings indicate that the Health-ITUES supports item-level customization while remaining suitable for multilingual translation and cultural adaptation.

The psychometric evaluation indicated very high internal consistency for the impact and usefulness subscales (Cronbach α>0.95). Although such values reflect strong reliability, they also suggest that some items may capture overlapping aspects of the same construct [42]. This interpretation is supported by the relatively strong interitem correlations observed within these subscales (*r*>0.85). Although these patterns suggest potential item redundancy and warrant future examination of item reduction or refinement, it is important to note that the Health-ITUES was intentionally designed to allow item-level customization while preserving conceptual comparability across contexts. The higher correlation between impact and perceived usefulness was also observed in previous studies [25,26,29]. Consistent with this, we retained all 20 items in the Japanese version without modifications or deletions.

From a theoretical perspective, this study reinforces the importance of usability as a multidimensional construct in digital health and demonstrates that the 4-factor structure of the Health-ITUES remains stable even when adapted to conversational AI contexts. Methodologically, this study highlights the feasibility of combining item-level customization with rigorous cross-cultural translation procedures while preserving conceptual equivalence. This approach provides a model for adapting usability instruments to rapidly evolving digital health technologies. To date, no other Japanese usability scale has demonstrated conceptual comparability while allowing flexible item-level customization tailored to specific use cases. The Japanese version of the Health-ITUES is expected to serve as a valuable tool for assessing the usability of mHealth and conversational AI applications as their use expands.

The overall mean Health-ITUES score in this study was 3.4 (SD 0.9), which was lower than that reported in previous studies [24,25]. Several contextual factors may explain this difference. Our evaluation used a chatbot that is still in the development phase, which may have influenced perceptions of quality and usability. In contrast, earlier studies assessed mature, widely used applications developed for long-term engagement by specific patient populations, such as medication adherence support for individuals with HIV [27] or symptom management for dysmenorrhea [25], which may have already achieved high user acceptance. Moreover, this study involved a single-use interaction based on a hypothetical scenario rather than a real-world health context, which may have contributed to the lower scores.

At the same time, these findings highlight a practical advantage of the Health-ITUES: it can be used not only to evaluate relatively mature digital health tools but also to assess prototypes and guide iterative improvement during early-stage development. Future evaluations of the same chatbot using the Health-ITUES under conditions of continued use may help clarify how perceived usability evolves as interactions between users and conversational AI systems accumulate over time. Based on user feedback, we are currently developing an improved version of the chatbot by enhancing its frequently asked question responses and refining error messages. Incorporating the Health-ITUES from the early stages of development may support user-centered improvements and provide an objective basis for monitoring usability changes over time.

From a practical standpoint, the availability of a validated Japanese version of the Health-ITUES enables more systematic and standardized usability evaluations of mHealth and conversational AI tools in Japan.

A key strength of this study was its robust sample size and the diversity of participants in terms of region and occupation. The sample size of 301 participants in our study exceeded the commonly used 10 times per item rule [32,34]. The mean score on the 15-item DHLS (2.8, SD 0.4) aligned with that of previous Japanese studies [37], and the distribution of conversational AI use showed no marked skew, supporting the appropriateness of the sample for usability assessment in this context.

### Limitations

This study had some limitations. First, participants were not actual patients or individuals currently experiencing health concerns; instead, we conducted the usability evaluation under a hypothetical scenario. Therefore, the results may not fully reflect real-world usage, and future research should assess its usability in clinical or personal health contexts. Second, the study population was limited to young women. Although we selected this demographic to align with the intended application of the mHealth chatbot, it is important to examine whether the Health-ITUES remains a valid usability assessment tool across other user groups in Japan, such as adult men, older adults, and individuals with chronic conditions, to enhance its generalizability.

### Conclusions

This study presented the first Japanese version of the Health-ITUES and provides initial evidence of its reliability and validity in the context of an STI-related chatbot. To our knowledge, no other Japanese usability scale for mHealth combines validated psychometric properties with the flexibility to be customized according to specific use cases. By preserving conceptual equivalence while enabling standardized, user-centered usability evaluation, the Japanese version of the Health-ITUES addresses a critical gap in digital health assessment in Japan. As conversational AI and mHealth applications continue to expand, the Japanese version of the Health-ITUES may serve as a valuable tool for supporting the development, evaluation, and implementation of effective and user-centered digital health interventions.

## Supplementary material

10.2196/90483 Multimedia Appendix 1 The descriptive statistics of the 20 items (mean, SD, skewness, kurtosis, and item-total correlations).

## Abbreviations

AVE: average variance extracted
CFA: confirmatory factor analysis
CFI: Comparative Fit Index
COSMIN: Consensus-Based Standards for the Selection of Health Measurement Instruments
CR: composite reliability
DHLS: Digital Health Literacy Scale
GFI: Goodness-of-Fit Index
Health-ITUEM: Health IT Usability Evaluation Model
Health-ITUES: Health Information Technology Usability Evaluation Scale
MARS: Mobile App Rating Scale
MAUQ: mHealth App Usability Questionnaire
mHealth: mobile health
PSSUQ: Post-Study System Usabiity Questionnaire
RMSEA: root mean square error of approximation
SRMR: standardized root mean square residual
STI: sexually transmitted infection
SUS: System Usability Scale
TAM: technology acceptance model
TLI: Tucker-Lewis Index
WHO: World Health Organization
