# Supplementary material for: Japanese Health Information Technology Usability Evaluation Scale for Sexually Transmitted Infection–Related Chatbots: Development and Psychometric Validation Study
Source: JMIR Hum Factors. 2026 Jul 23;13:e90483. doi: 10.2196/90483 (PMC13394856; doi:10.2196/90483)
Supplement: Multimedia Appendix 1 [file humanfactors-v13-e90483-s001.docx]

**Appendix Table 1**. The descriptive statistics of the 20 items (mean, standard deviation, skewness, kurtosis, and item–total correlations)

| Item number | mean | SD | Skewness | Kurtosis | Corrected Item- Total Correlation |
| --- | --- | --- | --- | --- | --- |
| 1 | 3.59 | 1.037 | -0.76 | 0.284 | 0.864 |
| 2 | 3.49 | 1.044 | -0.685 | 0.156 | 0.880 |
| 3 | 3.48 | 1.094 | -0.674 | 0.002 | 0.863 |
| 4 | 3.53 | 1.047 | -0.742 | 0.194 | 0.867 |
| 5 | 3.5 | 1.035 | -0.649 | 0.064 | 0.869 |
| 6 | 3.5 | 1.041 | -0.655 | 0.087 | 0.861 |
| 7 | 3.47 | 1.047 | -0.644 | 0.025 | 0.862 |
| 8 | 3.46 | 1.024 | -0.655 | 0.099 | 0.842 |
| 9 | 3.33 | 1.117 | -0.518 | -0.359 | 0.857 |
| 10 | 3.39 | 1.038 | -0.563 | 0.026 | 0.853 |
| 11 | 3.42 | 1.025 | -0.621 | 0.123 | 0.867 |
| 12 | 3.46 | 1.04 | -0.666 | 0.114 | 0.879 |
| 13 | 3.24 | 1.193 | -0.443 | -0.666 | 0.810 |
| 14 | 3.64 | 1.097 | -0.729 | 0.018 | 0.791 |
| 15 | 3.53 | 1.115 | -0.674 | -0.071 | 0.805 |
| 16 | 3.29 | 1.164 | -0.46 | -0.505 | 0.834 |
| 17 | 3.51 | 1.038 | -0.661 | 0.136 | 0.637 |
| 18 | 3.37 | 1.131 | -0.449 | -0.367 | 0.513 |
| 19 | 3.19 | 1.073 | -0.295 | -0.281 | 0.715 |
| 20 | 3.17 | 1.079 | -0.381 | -0.292 | 0.723 |
